# Report of Three Cases of Hydrocephalus1Read before the obstetric section of the Buffalo Academy of Medicine, October 12, 1897.

**Published:** 1897-11

**Authors:** L. Schröter

**Affiliations:** Buffalo, N. Y.


### Clinical Report.

### REPORT OF THREE CASES OF HYDROCEPHALUS.[1]

By L. SCHRÖTER, M. D., Buffalo, N. Y.

I REPORT my cases not on account of their special interest or that I can give you any new observations on the subject, but owing to the comparative rarity of true fetal hydrocephalus—one case occurring in about 3,000 labors. This and the gravity of the situation, when we are brought face to face with it, permits me to offer an excuse for calling your attention to such an old subject.

Case I.—The first case I saw was in 1890; the woman, a multipara, with nothing of interest in her history; she was in labor twenty hours, pains were very strong, membranes ruptured several days before; a midwife was in attendance. Before I was called to the case another physician attempted forceps for over an hour. I found os dilated, could feel the head at the brim, but could not determine the position. I applied forceps, but they slipped off every time I made traction; I then anesthetised the woman, introduced the whole hand in order to map out the position and then it was clear to me that it was a case of hydrocephalus, the sutures being far apart and the fontanelles very wide. On the palm of the examining hand I introduced a pair of long scissors and perforated the head; a gush of water corroborated the diagnosis. With the index and middle finger in the perforation opening I extracted the head without any difficulty. The woman suffered for many weeks with puerperal infection, also very extensive sloughing and ultimately succumbed.

Case II.—In 1891, Mrs. J., attended by a midwife in her third labor; breech presentation; midwife called me an hour after the extremities and trunk had been born. She tried all she could, but the head would not follow. I was not more successful than the midwife in my attempts to deliver manually the after-coming head, and having exhausted all the skill I possessed and all my patience, I decided to perforate, but it was impossible to reach any yielding spot, so I had to work my way through the foramen occipital magnum. To my great surprise, instead of brain tissue there came water in great quantity and the head followed on the very lightest traction. After the child had been washed the midwife directed my attention to a growth on his back. It was not hard for one to recognise a hydrorrhachis, which I did not notice earlier, because the child's back was lying posteriorly; furthermore, it was concealed from view by the towel I had around it. Another thing escaped my atten-

1. Read before the obstetric section of the Buffalo Academy of Medicine, October 12, 1897.

tion which might have aided me in arriving at a diagnosis—namely, I told the midwife to exert pressure on the head, I being unable to do it, as I had one hand on the nape of the neck, while the fingers of the other were making traction on the jaw ; had I been able to feel the head myself its extraordinary size could not have escaped my attention. The convalescence was uneventful.

Case III.—I saw in November, 1896, a woman, a multipara, 35 years old, whose history was negative. She was in labor only four hours, attended by her mother ; it was a footling ; the case was normal until the arms were born ; the head would not follow, notwithstanding the efforts at traction made by several neighbor-women. I was called after three hours and found the woman quite comfortable, pains having stopped shortly after the arms had been born. The body of the child furnished sufficient proof of rough handling, which had lasted for over an hour, being very much bruised and of a deep blue color ; on the back, in the lower lumbar region, there was a tumor the size of a lemon, of red wine color. I recognised that I had a case of spina bifida, with the possibilities of hydrocephalus. On examining I could feel a very soft spot, reminding one of the amniotic sac, which I punctured with a long pair of curved scissors, and later I saw that it was the mastoid fontanelle which I had perforated. The hydrocephalic fluid, that I caught in a vessel prepared for this purpose, measured over two quarts. The frontoöccipital circumference of the head was twenty-one inches. The patient recovered rapidly.

I might have, in this case, opened the hydrorrhachic sac and drained off the fluid, as advised by Tarnier, who has done it several times, but as the perforation of the head did not present any difficulty I chose this procedure, although I admit that draining through the spinal canal is to be preferred in identical cases, as it is much safer for the mother.

Having described my cases in a general way, I think it will be of interest to relate a few facts about hydrocephalus. We are unacquainted with the etiology of this anomaly, but our knowledge of it is of no practical importance, as it could hardly be prevented. However, of the greatest importance is an early diagnosis. An extremely large and rounded head must arouse our suspicion, which will be corroborated if we feel for the sutures and fontanelles, which are very wide; sometimes fluctuation may even be felt. The main difficulty in arriving at a diagnosis is that, as the head does not engage in most of the cases, we examine only a very small area of it and we may not meet a single suture; therefore, whenever in doubt, it is best to examine with the whole hand—a good general rule, by the way, in making diagnoses in difficult obstetric cases—and then we cannot fail to grasp the situation. It is almost impossible to make the diagnosis when the child presents itself with breech, foot or transversely and, to make the thing worse, breech presentations are much more frequent in cases of hydrocephalus than normally ; here the condition can only be presumed and the thing to guide us is the extraordinary size of the head as it is felt through the uterine walls ; or, in some cases, spina bifida, if present, should direct our attention to it, as these two anomalies are quite often combined.

As if to counterbalance this difficulty of diagnosis, the danger of hydrocephalus of the after-coming head is less than in head presentation, as the pressure never will last so long. The physician, seeing the unsurmountable obstacle in extracting the head and knowing the child dead, will perforate and thus through an accurate therapy he will correct a wrong diagnosis. The difference in the prognosis in head and breech (resp. foot) presentations is best illustrated by Schuchard's statistics, where, out of seventy-three cases of hydrocephalus, rupture of the uterus occurred fourteen times (eight spontaneously) and all in head presentations. The prognosis is sometimes more favorable in higher degrees of hydrocephalus, on account of the greater facility in molding of the elastic membranous skull. In exceptional cases the internal hydrocephalus may be transformed into an external and thus the head will pass the pelvis without any difficulty. It is proven by statistics that 25 per cent. of women die from rupture of the uterus, the cause of this misfortune being the same as of rupture in neglected transverse presentations. In exceptional cases it may happen that the hydrocephalic head will rupture spontaneously. More interesting are cases like those reported by Professor Fochier in the Obstetrical Society of France (April, 1895). In one of his cases "the head presented, but did not come down, when suddenly a large amount of fluid was discharged, after which the head came down and was delivered. It was then found that a spina bifida had broken, the liquid, which filled up the cranial cavity, escaping through the opening thus made." In another case, similar to the preceding, the spinal tumor was also ruptured, giving issue to hydrocephalic fluid. The margins of the tumor were sutured by Fochier ; the spina healed and the hydrocephalus was not reproduced.

The therapy in cases of hydrocephalus must be adapted to the special indications. Very seldom we will be able to save the life of the child, but we must attempt to do it in every case and, therefore, we should not perforate, but puncture the head with a trocar. Cases are reported in which living children were born after such manipulation. Under no circumstances should forceps be applied to a hydrocephalic head, as it will undoubtedly slip off in every case, the curve of the forceps not corresponding to the curvature of a hydrocephalic head, the blades being too short and the bones too elastic. Even after the head has been tapped, according to Carl Schröder, podalic version is to be preferred to forceps, when speedy delivery is indicated, but I think there is too much danger in such a procedure; the cervix and vagina have been too extensively stretched by the hydrocephalic head and the attempt to turn may be the last stimulus in producing the rupture of the womb.

529 Fillmore Avenue.

